# Dendrimer‐Stabilized Gold Nanoflowers Embedded with Ultrasmall Iron Oxide Nanoparticles for Multimode Imaging–Guided Combination Therapy of Tumors

**DOI:** 10.1002/advs.201801612

**Published:** 2018-11-12

**Authors:** Shiyi Lu, Xin Li, Jiulong Zhang, Chen Peng, Mingwu Shen, Xiangyang Shi

**Affiliations:** ^1^ State Key Laboratory for Modification of Chemical Fibers and Polymer Materials College of Chemistry Chemical Engineering and Biotechnology Donghua University Shanghai 201620 P. R. China; ^2^ Cancer Center Shanghai Tenth People's Hospital School of Medicine Tongji University Shanghai 200072 P. R. China

**Keywords:** combination therapy, dendrimers, gold nanoflowers, multimode imaging, ultrasmall iron oxide nanoparticles

## Abstract

Development of multifunctional theranostic nanoplatforms with improved diagnostic sensitivity and therapeutic efficiency of tumors still remains a great challenge. A unique multifunctional theranostic nanoplatform based on generation 5 (G5) poly(amidoamine) dendrimer–stabilized gold nanoflowers (NFs) embedded with ultrasmall iron oxide (USIO) nanoparticles (NPs) for multimode *T*_1_‐weighted magnetic resonance (MR)/computed tomography (CT)/photoacoustic (PA) imaging–guided combination photothermal therapy (PTT) and radiotherapy (RT) of tumors is reported here. G5 dendrimer–stabilized Au NPs and citric acid–stabilized USIO NPs are separately prepared, the two particles under a certain Fe/Au molar ratio are mixed to form complexes, the complexes are exposed to Au growth solution to form NFs via a seed–mediated manner, and the remaining dendrimer terminal amines are acetylated. The formed dendrimer‐stabilized Fe_3_O_4_/Au NFs (for short, Fe_3_O_4_/Au DSNFs) have a mean diameter of 99.8 nm, display good colloidal stability and cytocompatibility, and exhibit a near‐infrared absorption feature. The unique structure and composition of the Fe_3_O_4_/Au DSNFs endows them with a high *r*_1_ relaxivity (3.22 mM^−1^ s^−1^) and photothermal conversion efficiency (82.7%), affording their uses as a theranostic nanoplatform for multimode MR/CT/PA imaging and combination PTT/RT of tumors with improved therapeutic efficacy, which is important for translational nanomedicine applications.

Nanoplatforms integrated with both diagnosis and therapeutic elements are essential for precision medicine applications.^1^ A variety of theranostic systems have been developed in recent years for imaging‐guided therapy of different diseases.^2^ Among them, magnetic Fe_3_O_4_ nanoparticles (NPs) have been used for magnetic resonance (MR) imaging due to their *T*_2_‐shortening effect,^3^ while Au NPs with a specific shape have been employed as excellent theranostic agents for computed tomography (CT) imaging and photothermal therapy (PTT) of tumors by virtue of their good X‐ray attenuation property and near‐infrared (NIR) absorption feature.[[qv: 3a,4]] To achieve dual mode MR/CT imaging for precision diagnostic applications, it is important to integrate both Fe_3_O_4_ NPs and Au NPs within one nanoplatform.[qv: 5] However, in most cases, magnetic Fe_3_O_4_ NPs with a size bigger than 5 nm have been incorporated within the composite system for *T*_2_‐weighted MR imaging. Since the resulting negative contrast MR images with a dark signal are difficult to be distinguished from lesions, tumors, and some hypointense areas (metal deposition, calcification and bleeding, etc.), the diagnosis based on *T*_2_ MR imaging may be misleading in some cases. Therefore, development of a theranostic nanoplatform with *T*_1_ MR imaging performance is essential to achieve the goal of precision diagnosis.^6^

In recent years, ultrasmall Fe_3_O_4_ NPs (for short, USIO NPs) with a size less than 5 nm have been proven to be one of the most promising positive contrast agents for *T*_1_‐weighted MR imaging due to their high *r*_1_ relaxivity.^7^ USIO NPs can be surface modified with different functionalities for improved tumor *T*_1_ MR imaging application.^7^, ^8^ In addition, Au NPs with specific shapes such as nanostars (NSs),^9^ nanorods (NRs),^10^ and nanoflowers (NFs)^11^ have been employed as excellent theranostic agents for photoacoustic (PA) imaging and PTT of tumors owing to their outstanding biocompatibility and high photothermal conversion efficiency.[[qv: 4a,12]] Although earlier studies performed in our group have shown that superparamagnetic iron oxide NPs can be integrated with Au NSs to form Fe_3_O_4_@Au core/shell NSs and they can be further modified with either hyaluronic acid[qv: 13] or folic acid^14^ for dual mode CT/*T*_2_‐weighted MR imaging and PTT of tumors, there are currently no literature reports relevant to the combination of Au NPs with NIR absorption feature with USIO NPs for theranostic applications.

Moreover, due to the advantages of Au NPs that possess negligible toxicity, radiosensitizing property, high degree of control on size and shape, and easy surface modification with various biomolecules and biopolymers, the Au‐based composite NPs can be used for combination therapy of tumors, which significantly improved the tumor treatment efficacy.^15^ For instance, Li et al. reported that the Au NS–coated hollow mesoporous silica spheres can be modified with polyethylene glycol (PEG) and arginine‐glycine‐aspartic acid peptide and encapsulated with anticancer drug doxorubicin for combination chemotherapy and PTT of tumors.^16^ Liu et al. synthesized PEGylated Au@Pt nanodendrites for CT imaging and photothermal/radiation synergistic therapy of tumors.^17^

To integrate USIO NPs within an Au nanostructure having an NIR absorption feature and surface functionality, it is important to design the seed particles. Earlier work has shown that Au NPs or Ag NPs can be used as seed particles to mediate the growth of Au NSs using Au growth solution.^13^, ^14^ In addition, we have demonstrated that Au NPs with a size of 10–20 nm can be prepared using dendrimers as stabilizers through a mild self‐reduction method.^18^ Hence, it is reasonable to hypothesize that by judicious design of the seed particles containing USIO NPs, composite Fe_3_O_4_@Au NPs with NIR property may be formed for multimode *T*_1_ MR/CT/PA imaging and combination PTT and radiotherapy (RT) of tumors.

In the current work, we present a facile approach to synthesize dendrimer‐stabilized Au NFs embedded with USIO NPs for multimode imaging–guided tumor combination therapy. In our work, we first prepared generation 5 (G5) poly(amidoamine) (PAMAM) dendrimer–stabilized Au NPs (Au DSNPs) and citrate‐stabilized USIO NPs by self‐reduction and hydrothermal method, respectively. Then, through 1‐(3‐dimethylaminopropyl)‐3‐ethylcarbodiimide hydrochloride (EDC)‐mediated covalent reaction, seed particles containing both Au DSNPs and USIO NPs were formed and used to generate Au NFs. The terminal amines of the PAMAM stabilizer were finally acetylated to shield the positive surface charge of the NFs (**Figure** **1**). The thus prepared dendrimer‐stabilized Au NFs (for short, Au DSNFs) embedded with USIO NPs were systematically characterized to delineate their structure, size and morphology, composition, *r*_1_ relaxivity, CT and PA imaging property, cytotoxicity, cellular uptake efficiency. Finally they were used for multimode *T*_1_ MR/CT/PA imaging and combination PTT and RT of a murine breast tumor model. To our best knowledge via a thorough literature investigation, our study is the first one relevant to the preparation of multifunctional Au DSNFs embedded with USIO NPs through seed particle design for *T*_1_ MR/CT/PA imaging and combination PTT and RT of tumors.

**Figure 1:**
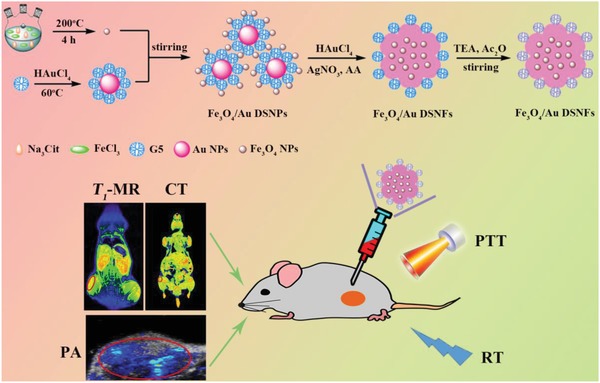
Schematic illustration of the preparation of the Fe_3_O_4_/Au DSNFs for multimode imaging–guided combination therapy of tumors.

To design seed particles, we first synthesized citrate‐stabilized USIO NPs according to the literature.^7^, ^8^ The formed USIO NPs displayed a diameter of 2.1 nm (Figure S1a,b, Supporting Information). Meanwhile, Au DSNPs with a Au core diameter of 12.2 nm (Figure S1c,d, Supporting Information) were prepared by a self‐reduction method using amine‐terminated G5 PAMAM dendrimers as a stabilizer according to the literature.^18^ The thermogravimetric analysis (TGA) data validated that the percentage of G5 dendrimers within the Au DSNPs was 83.2% (Figure S2, Supporting Information). After that, EDC‐activated USIO NPs were reacted with Au DSNPs via an amide linkage to form composite Fe_3_O_4_/Au NPs as seeds (Fe_3_O_4_/Au DSNPs) with different molar ratios of Fe/Au. We used zeta‐potential and dynamic light scattering (DLS) to characterize the prepared USIO NPs, Au DSNPs, and seed particles (Table S1, Supporting Information). Clearly, USIO NPs and Au DSNPs display a surface potential of −26.8 and 24.6 mV due to the presence of stabilizers of citric acid and PAMAM terminal amines, respectively. After formation of the seeds of Fe_3_O_4_/Au NPs with different Fe/Au molar ratios, their surface potentials are still slightly positive, indicating the neutralization of the dendrimer terminal amines. The hydrodynamic size of the Fe_3_O_4_/Au NPs was gauged to be within the range of 26–28 nm, which is smaller than the individual Au DSNPs, suggesting the strong interaction between Au DSNPs and USIO NPs. We note that the hydrodynamic sizes of USIO NPs and Au DSNPs measured by DLS are much larger than those determined by transmission electron microscopy (TEM). This could be because TEM measures single core Fe_3_O_4_ and Au NPs, whereas DLS determines the hydrated particles in a possibly clustered structure having slight agglomeration, in accordance with the literature data.^19^ It is also interesting to be noted that we mixed both USIO NPs and Au DSNPs at different Fe/Au molar ratios in the presence of EDC/*N*‐hydroxysuccinimide (NHS), this does not exclude the possibility for them to form complexes via electrostatic interaction or other weak forces. Hence, the composition of USIO NPs and Au DSNPs should remain identical to the initial feeding ratio. This will be convenient for us to tune the composition of the seeds to get the optimized Fe_3_O_4_/Au DSNFs.

Taking the seeds of Fe_3_O_4_/Au DSNPs formed at Fe/Au molar ratio of 4:1 as an example, we demonstrated the composition of Fe_3_O_4_/Au DSNPs by elemental mapping and dark‐field TEM images (Figure S3a, Supporting Information), where the Fe (green spot) and Au (red spot) elements of Fe_3_O_4_/Au DSNPs were well distributed. Energy‐dispersive X‐ray spectroscopy (EDS) further illustrated the existence of both Fe and Au within the seed particles (Figure S4a, Supporting Information). To prove the crystalline structure of the seeds, X‐ray diffraction (XRD) pattern showed the typical diffraction peaks that are respectively associated to the Au NPs and USIO NPs (Figure S3b, Supporting Information). And the crystalline structure of Fe_3_O_4_/Au DSNPs was further verified by high‐resolution TEM (Figure S5, Supporting Information), where individual crystals of Au and USIO NPs can be found and the lattice spacings correspond well with the respective crystals. Particularly, the interplanar crystal spacing of Au and Fe_3_O_4_ crystals were observed and calculated to be 2.153 and 2.648 Å, respectively. In addition, selected area electron diffraction (SAED) was further used to confirm the composition and crystalline structure of the seed particles (Figure S6a, Supporting Information), where the typical rings belonging to the planes of cubic inverse spinel structure of USIO NPs and the face‐centered cubic (*fcc*) structure Au DSNPs can be found.

Next, the seed particles of Fe_3_O_4_/Au DSNPs (Fe/Au molar ratio = 4:1) were exposed to an Au growth solution to generate Fe_3_O_4_/Au DSNFs according to protocols reported in the literature.^13^, ^14^ The formed NFs were lastly subjected to acetylation to shield the remaining primary amines of G5 dendrimers, which can be confirmed by proton nuclear magnetic resonance spectroscopy (^1^H NMR) (Figure S7, Supporting Information) by the appearance of the acetyl protons at 1.90 ppm. It can be seen that the Fe_3_O_4_/Au DSNFs exhibit a nice flower structure with a narrow size distribution (**Figure** **2**a). The mean diameter of the NFs was determined to be 99.8 nm (Figure 2b). The composition of the NFs was clarified by elemental mapping (Figure 2c) and EDS (Figure S4b, Supporting Information). Clearly, the USIO NPs are well distributed within the Fe_3_O_4_/Au DSNFs, severe aggregation of the USIO NPs does not seem to appear. This will be particularly beneficial for the USIO NPs to be accessible to water protons for non‐compromised MR imaging applications. Furthermore, the crystalline structure of Fe_3_O_4_/Au DSNFs was validated by XRD (Figure 2d) and SAED pattern (Figure S6b, Supporting Information), quite similar to the seed particles, and the XRD peaks of USIO NPs are not prominent, in agreement with the literature.^7^ The major difference is that the Au‐associated peaks are much more prominent due to the growth of Au NFs with additional more Au amount loaded within the structure. It is clear that some peaks related to USIO NPs are not prominent, which is due to their ultrasmall size and again the relative small amount of the Fe when compared to Au.

**Figure 2:**
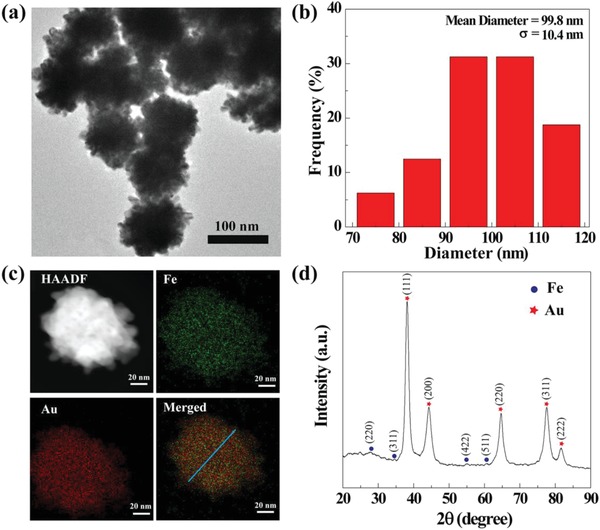
Structural characterization of Fe_3_O_4_/Au DSNFs. a) TEM images, b) size distribution histograms, c) element mapping, and d) XRD pattern of Fe_3_O_4_/Au DSNFs.

To optimize the structure and property of Fe_3_O_4_/Au DSNFs, we used the Fe_3_O_4_/Au DSNPs having different Fe/Au molar ratios (from 1:1 to 6:1) as seed particles to synthesize NFs and tested the colloidal stability, size and morphology, surface potential, and NIR absorption properties of the NFs. We show that the synthesized NFs are stable at the Fe/Au molar ratios from 1:1 to 4:1, and the precipitation occurs at the Fe/Au molar ratio up to 6:1 after one week storage at room temperature (Figure S8, Supporting Information). The synthesized Fe_3_O_4_/Au DSNFs using seeds having different Fe/Au molar ratios (from 1:1 to 4:1) were also characterized by zeta potential and DLS measurements (Table S1, Supporting Information). In all cases, the formed NFs have a surface potential of 14–17 mV, similar to Fe‐free Au DSNFs. However, the hydrodynamic sizes are in the range of 193–302 nm, which is much larger than Fe‐free NFs, likely due to the incorporation of USIO NPs.

After that, the NIR absorption feature of different Fe_3_O_4_/Au DSNPs and Fe_3_O_4_/Au DSNFs was recorded via UV–vis spectrometry. Clearly, the formed Fe_3_O_4_/Au DSNPs (**Figure** **3**a) have a localized surface plasmon resonance (LSPR) peak at 522 nm and the intensities of LSPR peak at 522 nm become more and more apparent with the increase of the Fe/Au molar ratio from 1:1 to 4:1. This suggests that at the fixed Au concentration, the addition of more USIO NPs renders the seed particles with increased LSPR peak intensity, likely due to the improved light exposure of the Au DSNPs in the seed particles. Similarly, after the formation of the Fe_3_O_4_/Au DSNFs using seeds of Fe_3_O_4_/Au DSNPs at different Fe/Au molar ratios, an NIR peak at 680–730 nm emerges and has a redshift when compared to Fe‐free Au DSNFs (670 nm). With the increase of Fe/Au molar ratio, the NIR absorption of Fe_3_O_4_/Au DSNFs has both increased absorption intensity and enlarged redshift (Figure 3b). This result indicates that the embedded USIO NPs in Fe_3_O_4_/Au DSNFs are beneficial to enhance the NIR absorption, and the Fe_3_O_4_/Au DSNFs prepared with seeds having an Fe/Au molar ratio of 4:1 are suitable for PA imaging and PTT of tumors under 808 nm laser irradiation due to their optimized NIR absorption feature.

**Figure 3:**
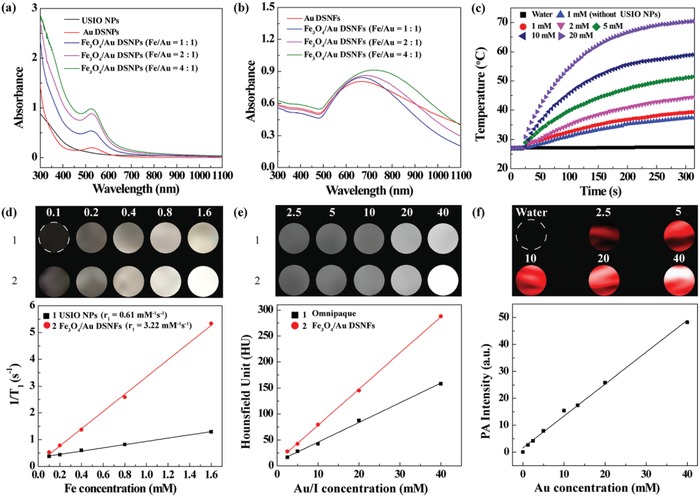
Optical, photothermal, and multimode imaging properties of the Fe_3_O_4_/Au DSNFs. a) UV–vis spectra of USIO NPs, Au DSNPs, and Fe_3_O_4_/Au DSNPs with different Fe/Au molar ratios of 1:1–4:1 ([Au] = 0.9 × 10^−3^ m). b) UV–vis spectra of Au DSNFs and Fe_3_O_4_/Au DSNFs with different Fe/Au molar ratios of 1:1–4:1 ([Au] = 0.9 × 10^−3^ m). c) Temperature elevation of water (as control) and the aqueous solution of Au DSNFs ([Au] = 1 × 10^−3^ m, for comparison) and Fe_3_O_4_/Au DSNFs at different Au concentrations under the 808 nm laser irradiation (1.2 W cm^−2^) as a function of irradiation time. d) *T*_1_‐weighted MR images and linear fitting of 1/*T*_1_ of Fe_3_O_4_ NPs and Fe_3_O_4_/Au DSNFs at different Fe concentrations (1 and 2 represents USIO NPs and NFs, respectively). e) CT images and CT value (HU) of Omnipaque and Fe_3_O_4_/Au DSNFs with different concentrations of radiodense element of Au or I (1 and 2 represents Omnipaque and NFs, respectively). f) PA images and PA values of Fe_3_O_4_/Au DSNFs with different Au concentrations.

With the remarkable NIR absorption feature, we then evaluated the photothermal conversion property of the USIO NP–embedded Au DSNFs (Figure 3c). The temperature of the aqueous solution containing Fe_3_O_4_/Au DSNFs at different Au concentrations under laser irradiation increases with the laser irradiation time and with the increase of Au concentration. The temperature increases from 27 °C to over 70 °C after laser irradiation for 300 s at the Au concentration of 20 × 10^−3^ m (Figure 3c). In sharp comparison, the temperature of pure water only displays a slight increase after laser irradiation. Interestingly, the Fe‐free Au DSNFs displays a Δ*T* of 10.4 °C, which is smaller than the Fe_3_O_4_/Au DSNFs (Δ*T* = 12.3 °C) at the same Au concentration (1 × 10^−3^ m), implying that the embedded USIO NPs is helpful to strengthen the photothermal conversion property. The Δ*T* of Fe_3_O_4_/Au DSNFs solution at different Au concentrations was quantified to be 12.3, 17.6, 24.8, 32.3, and 43.5 °C for [Au] at 1 × 10^−3^, 2 × 10^−3^, 5 × 10^−3^, 10 × 10^−3^, and 20 × 10^−3^ m, respectively (Figure S9a, Supporting Information). In addition, the temperature of Fe_3_O_4_/Au DSNFs could reach over 45 °C from initial 27 °C after five cycles of heating/cooling under laser irradiation (Figure S9b, Supporting Information), suggesting that the Fe_3_O_4_/Au DSNFs possess an excellent photothermal stability.

Next, the phototermal conversion efficiency (η) of the Fe_3_O_4_/Au DSNFs was measured to be 82.7% according to the method reported in the literature (Figure S9c,d, Supporting Information),^20^ which is much higher than that of the pure Au DSNFs (*η* = 63.1%) (Figure S10, Supporting Information) and those reported in the literature.^9^, ^16^, ^21^ This is likely because the doped high amount of USIO NPs within the Fe_3_O_4_/Au DSNFs may play a synergistic reinforcing role by increasing the surface area of the Au NFs. The thermal images (Figure S11, Supporting Information) of the Fe_3_O_4_/Au DSNFs under laser irradiation further validated the photothermal conversion property of the Fe_3_O_4_/Au DSNFs.

To prove the multimode *T*_1_‐weighted MR/CT/PA imaging properties, we performed in vitro phantom studies (Figure 3d–f). For MR imaging, both USIO NPs and the Fe_3_O_4_/Au DSNFs can make the MR imaging contrast enhanced in an Fe concentration‐dependent manner (Figure 3d), and the *r*_1_ relaxivity of the Fe_3_O_4_/Au DSNFs (3.2 mM^−1^ s^−1^) is much higher than that of the free USIO NPs (0.61 mM^−1^ s^−1^). Meanwhile, the signal intensity of *T*_1_ MR images of Fe_3_O_4_/Au DSNFs is much higher than that of USIO NPs at the same Fe concentrations (Figure 3d). The increased *r*_1_ relaxivity of the Fe_3_O_4_/Au DSNFs could be due to the fact that the used G5 dendrimer‐stabilized Au NPs are able to sufficiently disperse USIO NPs, and the formation of NFs via seed‐mediated method does not seem to induce significant aggregation of USIO NPs. This is significantly different from that reported in the literature related to the association of USIO NPs with pure dendrimers that leads to decreased *T*_1_ MR imaging effect of USIO NPs.^22^ Meanwhile, due to the flower structure, the accessibility of water protons to the surface of USIO NPs does not seem to be compromised.

In addition, the CT imaging property of Fe_3_O_4_/Au DSNFs was revealed by comparing with Omnipaque, a clinically used CT contrast agent (Figure 3e). The brightness of CT images and the CT value of both Fe_3_O_4_/Au DSNFs and Omnipaque increase with the Au or I concentration, and at the same radiodense element concentration (Au or I), Fe_3_O_4_/Au DSNFs display much higher CT value than Omnipaque. Furthermore, due to the NIR absorption feature, the Fe_3_O_4_/Au DSNFs display PA imaging contrast enhancement with the Au concentration and the PA value is in a linear correlation with the Au concentration. Hence, the designed DSNFs may be potentially used for multimode *T*_1_ MR/CT/PA imaging and PTT of tumors.

To evaluate the intrinsic cytocompatibility of the prepared Fe_3_O_4_/Au DSNFs, CCK‐8 assay was performed (**Figure** **4**a). The viability of 4T1 cells treated with the Fe_3_O_4_/Au DSNFs slightly decreases with the increase of Au concentration and the overall viability remains above 94% at the highest Au concentration tested (2.0 × 10^−3^ m), suggesting that the Fe_3_O_4_/Au DSNFs possess an excellent cytocompatibility in the given concentration range. To check the cellular uptake ability of the particles, the Au uptake within the 4T1 cells treated with the Fe_3_O_4_/Au DSNFs for 6 h was quantitatively analyzed by inductively coupled plasma‐optical emission spectroscopy (ICP‐OES) (Figure S12, Supporting Information). The Au uptake within the 4T1 cells is concentration‐dependent, and the cellular Au uptake amount can reach about 10.2 pg cell^−1^ at the maximum Au concentration of 2.0 × 10^−3^ m studied. In addition, qualitative observation of the cellular Fe uptake was also confirmed by Prussian blue staining of cells (Figure S13, Supporting Information), where more blue staining can be observed after the cells were treated with the Fe_3_O_4_/Au DSNFs with the increase of Fe concentration. The results validate that the Fe_3_O_4_/Au DSNFs are able to be taken up by cells likely via pathways of phagocytosis and diffusion via cell walls.^23^

**Figure 4:**
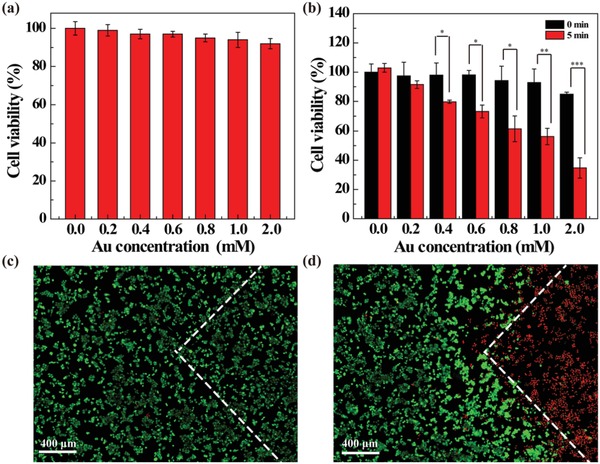
Cytocompatibility and photothermal ablation of cancer cells. a) CCK‐8 assay of 4T1 cell viability after treatment with Fe_3_O_4_/Au DSNFs at different Au concentrations for 24 h. b) CCK‐8 assay of 4T1 cell viability after treatment with Fe_3_O_4_/Au DSNFs at different Au concentrations for 6 h, followed by 808 nm laser irradiation for 0 and 5 min and then incubation of cells with CCK‐8 for 3 h. Fluorescence microscopic images of Calcein AM/PI‐stained 4T1 cells treated with c) PBS and d) Fe_3_O_4_/Au DSNFs ([Au] = 2 × 10^−3^ m) under 808 nm laser irradiation for 5 min. The white dotted line shows the margin of the laser facula region.

In order to investigate the effectiveness of PTT of cancer cells in vitro, the viability of 4T1 cells incubated with Fe_3_O_4_/Au DSNFs at different Au concentrations (0–2.0 × 10^−3^ m) without/with 808 nm laser exposure (1.2 W cm^−2^, 5 min) was measured by CCK‐8 assay (Figure 4b). Compared to cells without laser irradiation, the cell viability decreased dramatically after laser irradiation when the Au concentration exceeds 0.4 × 10^−3^ m. At the Au concentration up to 2.0 × 10^−3^ m, the cell viability decreases to 34.7% under laser irradiation, which is significantly lower than that without laser irradiation (85%, *p* < 0.001). Furthermore, the photothermal therapeutic effect of the Fe_3_O_4_/Au DSNFs for 4T1 cells was confirmed by fluorescence microscopic observation (Figure 4c,d). The cells treated with Fe_3_O_4_/Au DSNFs without laser irradiation show regular cell morphology with green fluorescence in the entire region, while the cells treated with Fe_3_O_4_/Au DSNFs under laser irradiation display obvious boundary of the green fluorescence for live cells and the red fluorescence for dead ones in the laser facula region. These results imply that the Fe_3_O_4_/Au DSNFs are able to exert photothermal ablation effect of cancer cells under laser irradiation.

Next, we explored the use of Fe_3_O_4_/Au DSNFs for multimode MR/CT/PA/thermal imaging of a subcutaneous tumor model in vivo (**Figure** **5**). Animal experiments were performed following the protocols approved by the animal care committee of Shanghai Tenth People's Hospital (with a license number of SYXK (Hu) 2014‐0026) and also the policy of the National Ministry of Health. Clearly, the MR signal‐to‐noise ratio (SNR) of tumor reaches the peak value at 60 min after intravenous injection of USIO NPs or Fe_3_O_4_/Au DSNFs, and then the MR SNR of tumor gradually decreases due to the metabolism of NPs in vivo after 60 min (Figure 5a_1_). It is worth noting that the mice treated with the Fe_3_O_4_/Au DSNFs display a clearer *T*_1_‐weighted MR images (Figure 5a) and higher MR SNRs in tumor region than those treated with the USIO NPs (Figure 5a_1_). Especially, at 60 min postinjection, the tumor SNR treated with the Fe_3_O_4_/Au DSNFs has a peak value around 1.4 times higher than that treated with USIO NPs (*p* < 0.001). These results demonstrate that the Fe_3_O_4_/Au DSNFs display synergistically enhanced *T*_1_ effect compared with USIO NPs.

**Figure 5:**
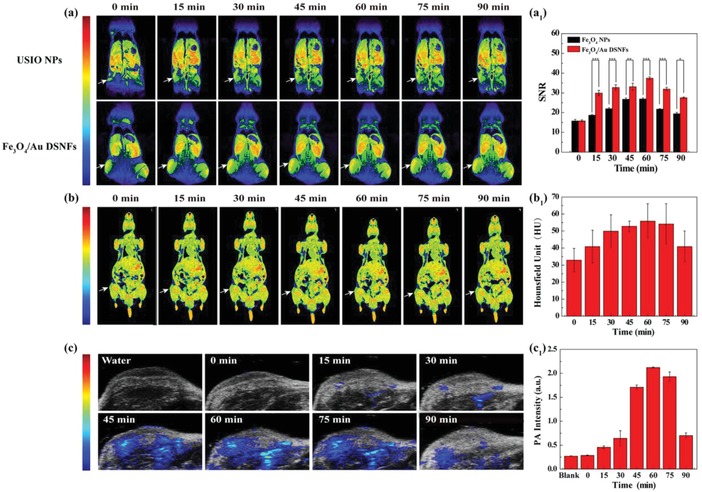
In vivo tumor multimode imaging. a) In vivo *T*_1_‐weighted MR images and a_1_) the corresponding MR SNR of the 4T1 tumor before injection and at different time points postinjection of USIO NPs and Fe_3_O_4_/Au DSNFs ([Fe] = 5.5 × 10^−3^ m, in 200 µL NS for each mouse), respectively (mean ± S.D., *n* = 3). The MR signal intensity of blank (air) was used as the background (noise). b) CT images and b_1_) corresponding CT values of the 4T1 tumor in nude mice before and at different time points post intravenous injection of the Fe_3_O_4_/Au DSNFs ([Au] = 0.04 m, in 200 µL NS for each mouse). c) PA images and c_1_) the corresponding PA values of 4T1 tumor in nude mice before and at different time points post intravenous injection of the Fe_3_O_4_/Au DSNFs ([Au] = 0.04 m, in 200 µL NS for each mouse) under 808 nm laser irradiation.

Similarly, for CT imaging (Figure 5b,b_1_), the tumor CT value reaches the peak value at 60 min after intravenous injection of Fe_3_O_4_/Au DSNFs, and the peak value (55.9 HU) is 1.7 times higher than that before injection (32.9 HU). It should be noted that the Fe_3_O_4_/Au DSNFs were well distributed in the whole body through blood circulation after intravenous injection, thus inevitably resulting in the retention of Fe_3_O_4_/Au DSNFs in the main organs of mice. Thus, for both MR and CT images, both the left and right legs of mice were lighted. Importantly, we have noted that the MR and CT signal intensities of tumor are much higher than the surrouding tissues. Due to the NIR absorption feature, the Fe_3_O_4_/Au DSNF enabled PA imaging of tumors with a peak PA value also at 60 min postinjection (Figure 5c,c_1_). Notably, the time point of peak value of MR, CT, and PA imaging intensity is consistent, suggesting that the Fe_3_O_4_/Au DSNFs might be accumulated within the tumor region through the passive enhanced permeability and retention (EPR) effect and the accumulation amount can reach the maximum at 60 min after intravenous injection. Overall, the Fe_3_O_4_/Au DSNFs were able to act as a contrast agent for multimode MR/CT/PA imaging of tumors in vivo.

Further, due to the photothermal conversion effect, the Fe_3_O_4_/Au DSNF also enabled thermal imaging of tumors after they were intratumorally injected (**Figure** **6**a,b). The temperature of tumor region (Bx1) treated with the Fe_3_O_4_/Au DSNFs under laser irradiation for 300 s increases rapidly to about 57 °C, while that (Bx1) treated with normal saline (NS) under laser irradiation hardly changes, further indicating that the Fe_3_O_4_/Au DSNFs are promising to be used for PTT of tumors.

**Figure 6:**
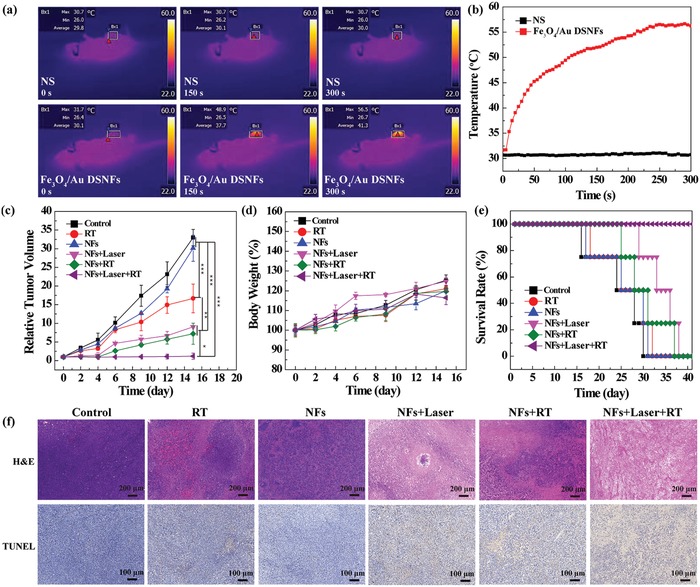
In vivo thermal image and combination therapy of tumor. a) Photothermal images and b) corresponding temperature profiles of 4T1 tumor–bearing mice after intratumoral injection of NS (0.1 mL for each mouse) or Fe_3_O_4_/Au DSNFs ([Au] = 20 × 10^−3^ m, in 0.1 mL NS for each mouse) under 808 nm laser irradiation at different time points, respectively. The c) relative tumor volume, d) body weight, and e) survival rate of 4T1 tumor–bearing mice as a function of time post treatment. f) H&E staining and TUNEL staining of tumor sections after different treatments.

As mentioned in our previous studies, PTT of tumors enables the increase of oxygen concentration in the blood flow of tumor region as well as the increased blood vessel permeability, which in turn is beneficial for sensitized RT of tumors.^21^ Hence, combination of RT and PTT is very effective to treat tumors. In this study, we also explored the synergistic therapeutic effect of combinational PTT and RT using Fe_3_O_4_/Au DSNFs for tumor treatment (Figure 6c–f). Clearly, the tumors in the control and NFs group are growing gradually with the time postinjection. In contrast, the tumors in the groups of RT, NFs + Laser, and NFs + RT exhibit different degrees of inhibition compared to the original tumors (*p* < 0.001). The tumors in the NFs + Laser + RT group were able to more significantly inhibit the tumor growth when compared to that in the groups of RT, NFs + Laser, and NFs + RT (*p* < 0.05). The tumors in NFs + Laser + RT group were able to be completely ablated after 4 days and the tumors did not reoccur during the experimental time period (Figure 6c). In addition, the body weight of the mice in NFs + Laser + RT group was well maintained, similar to the other groups (Figure 6d), implying that all the treatments negligibly exert side‐effects during the therapeutic process. The survival rate of the mice in different groups was recorded to further illustrate the synergistic therapeutic efficacy of PTT and RT (Figure 6e). The mice in control, RT, and NFs group were all dead on the day 30 to day 32, and the mice in NFs + Laser and NFs + RT group were all dead on day 37 to day 38, respectively. In contrast, the survival rate of the mice in NFs + Laser + RT group is still 100% after 40 days.

Furthermore, H&E and TUNEL staining of the tumor sections in different groups was further used to confirm the synergistic therapeutic efficacy of PTT and RT (Figure 6f). The H&E staining of tumors in NFs + Laser, NFs + RT, and NFs + Laser + RT groups display large area of necrotic cells with positive staining, while rare necrotic cells with positive staining could be seen in the tumors of control and NFs groups. TUNEL staining of tumors in different groups shows that the tumor cell apoptosis region follows the order of NFs + Laser + RT (82.5%) > NFs + RT (56.6%) > NFs + Laser (42.7%) > RT (12.8%) > NFs (3.4%) > Control (3.1%) groups, which agrees well with the quantitative analysis of the cell apoptosis rate (Figure S14, Supporting Information). These results imply that the treatment of Fe_3_O_4_/Au DSNFs under laser irradiation followed by RT enables a synergetic‐enhanced therapy of tumors in vivo. In addition, the H&E staining of main organs (heart, liver, spleen, lung, and kidney) in the mice were further checked to evaluate the biosafety of Fe_3_O_4_/Au DSNFs (Figure S15, Supporting Information). The main organs in the mice treated with the Fe_3_O_4_/Au DSNFs show a similar profile comparable to those of the mice treated with phosphate buffered saline (PBS). These results suggest that the Fe_3_O_4_/Au DSNFs are harmless to those organs and display an excellent biosafety.

We further investigated the biodistribution of Au element to assess the metabolism of the Fe_3_O_4_/Au DSNFs at different time points post intravenous injection of the particles (Figure S16, Supporting Information). Clearly, at the earlier time point of 1 h postinjection, Au amount in tumor (38.6 µg g^−1^), liver (230.0 µg g^−1^), and spleen (209.2 µg g^−1^) are relatively high, indicating that the particles are able to quickly clear via the reticuloendothelial system (RES), and a significant part of the particles are able to escape the RES uptake, enabling effective multimode MR/CT/PA imaging of tumors. With the time postinjection, the Au uptake in the tumor, liver, spleen, and lung gradually decreases, and at 96 h postinjection, the particles are almost completely cleared out of all major organs, quite close to the control mice treated with PBS. These results fully illustrate that the Fe_3_O_4_/Au DSNFs can be eventually cleared out of body and have a good safety profile.

In summary, we developed a unique theranostic nanoplatform based on Fe_3_O_4_/Au DSNFs for multimode *T*_1_–weighted MR/CT/PA imaging and combination PTT/RT of tumors. Through the judicious design of seed particles composed of Au DSNPs and USIO NPs, Au NFs embedded with USIO NPs are able to be formed with uniform distribution of USIO NPs thanks to the role played by the dendrimer stabilizer. With the embedment of USIO NPs, the designed Fe_3_O_4_/Au DSNFs display a much higher *r*_1_ relaxivity (3.22 mM^−1^ s^−1^) than free USIO NPs, and enhanced NIR absorption property, hence having improved photothermal conversion efficiency (82.7%). With the further CT and PA imaging property, good colloidal stability, cytocompatibility, and cellular uptake efficiency, the designed Fe_3_O_4_/Au DSNFs are able to be used for MR/CT/PA imaging and combination PTT/RT of tumors. The designed Fe_3_O_4_/Au DSNFs may be used as a theranostic nanoplatform for multimode imaging–guided combination therapy of other types of tumors for translational nanomedicine applications.

## Conflict of Interest

The authors declare no conflict of interest.

## Supporting information

Supplementary
