# Supplementary material for: Dendrimer‐Stabilized Gold Nanoflowers Embedded with Ultrasmall Iron Oxide Nanoparticles for Multimode Imaging–Guided Combination Therapy of Tumors
Source: Adv Sci (Weinh). 2018 Nov 12;5(12):1801612. doi: 10.1002/advs.201801612 (PMC6299682; doi:10.1002/advs.201801612)
Supplement: Supplementary file 1 — Supplementary [file ADVS-5-1801612-s001.pdf]

Copyright WILEY-VCH Verlag GmbH & Co. KGaA, 69469 Weinheim, Germany, 2018.

ADVANCED SCIENCE
Open Access

# Supporting Information

for *Adv. Sci.,* DOI: 10.1002/advs.201801612

# Dendrimer-Stabilized Gold Nanoflowers Embedded with Ultrasmall Iron Oxide Nanoparticles for Multimode Imaging–Guided Combination Therapy of Tumors

*Shiyi Lu, Xin Li, Jiulong Zhang, Chen Peng,* Mingwu Shen,* and Xiangyang Shi**

Copyright WILEY-VCH Verlag GmbH & Co. KGaA, 69469 Weinheim, Germany, 2013.

# Supporting Information

**Dendrimer-Stabilized Gold Nanoflowers Embedded with Ultrasmall Iron Oxide Nanoparticles for Multimode Imaging-Guided Combination Therapy of Tumors**

Shiyi Lu, Xin Li, Jiulong Zhang, Chen Peng,* Mingwu Shen,* Xiangyang Shi*

Miss. S. Lu,[+] Mr. X. Li,[+] Prof. X. Shi, Prof. M. Shen
State Key Laboratory for Modification of Chemical Fibers and Polymer Materials, College of Chemistry, Chemical Engineering and Biotechnology, Donghua University, Shanghai 201620, P. R. China
E-mail: xshi@dhu.edu.cn (X. Shi), mwshen@dhu.edu.cn (M. Shen)

Mr. J. Zhang, Prof. C. Peng
Cancer Center, Shanghai Tenth People's Hospital, School of Medicine, Tongji University, Shanghai 200072, P. R. China
E-mail: pengchen_1985@163.com (C. Peng)

[+] Authors contributed equally to this work.

**Keywords:** gold nanoflowers; dendrimers; ultrasmall iron oxide nanoparticles; multimode imaging; combination therapy of tumors

**Experimental**

Materials

Ethylenediamine core G5.$NH_2$ PAMAM dendrimers were purchased from Dendritech (Midland, MI). 1-(3-Dimethylaminopropyl)-3-ethylcarbodiimide hydrochloride (EDC· HCl) and N-hydroxysuccinimide (NHS) were from GL Biochem. (Shanghai, China). Iron(III) chlorideanhydrous, $HAuCl_4$· $4H_2O$, silver nitrate ($AgNO_3$), ascorbic acid (AA), dimethyl sulfoxide (DMSO), diethylene glycol (DEG), trisodium citrate dihydrate ($Na_3Cit$· $2H_2O$), sodium acetate anhydrous (NaOAc) and other agents were from Sinopharm Chemical Reagent Ltd. (Shanghai, China). 4T1 cells (4T1 murine breast cancer cells) were obtained from Institute of Biochemistry and Cell Biology, the Chinese Academy of Sciences (Shanghai, China). Cell Counting Kit-8 (CCK-8) was purchased from 7Sea Pharmatech Co., Ltd. (Shanghai, China). Fetal bovine serum (FBS), Dulbecco's modified Eagle's medium (DMEM), penicillin, and streptomycin were obtained from Hangzhou Jinuo Biomedical Technology (Hangzhou, China). Cellulose dialysis membranes having a molecular weight cut-off (MWCO) of 3 000 were acquired from Shanghai Yuanye Biotechnology Corporation (Shanghai, China). Water used in all experiments was purified using a Milli-Q Plus 185 water purification system (Millipore, Bedford, MA) with a resistivity higher than 18.2 MΩ· cm.

Synthesis of USIO NPs

USIO NPs were synthesized according to our previous work.[1] In brief, $FeCl_3$ (4 mmol) was dissolved into 40 mL of diethylene glycol (DEG) under stirring and then $Na_3Cit$· $2H_2O$ (1.6 mmol) was added into the above solution at 80 $^{o}$C and reacted for 2 h, followed by addition of NaOAc (12 mmol) to form a clear solution. After that, the mixture was transferred to a stainless-steel reaction kettle with a volume of 50 mL and reacted at 200 $^{o}$C for 4 h. Afterwards, the black product was cooled down to ambient temperature, collected by

centrifugation (8 500 rpm, 15 min), and purified with anhydrous ethanol for 4 times. The resulting precipitate was dried at 60 $^oC$ and stored at -20 $^oC$ before use.

Synthesis of Au DSNPs

Au DSNPs were prepared according to the literature.[2] In brief, G5 dendrimers (0.002 mmol) were dissolved in water (30 mL) and the solution was preheated in a water bath at 60 $^oC$ for 30 min. Then, $HAuCl_4 \cdot 4H_2O$ (0.073 M, in 353 μL water) was added into the above solution under magnetic stirring at 60 $^oC$ for 3 h. The color of the reaction mixture gradually changed from auratus to lilac. Afterwards, the solution was cooled down to room temperature and lyophilized to get the product of Au DSNPs.

Formation of Complexes of $Fe_3O_4$/Au DSNPs with Different Fe/Au Molar Ratios

The surface carboxyl groups of USIO NPs (56 mg, in 5 mL DMSO) were activated by EDC (144 mg, in 2 mL DMSO) and NHS (70 mg, 2 mL DMSO) for 3 h under stirring. After activation, the product was dialyzed against water (6 times, 2 L) using a dialysis membrane with an MWCO of 3000 for 3 days, followed by lyophilization to obtain the product of carboxyl-activated ultrasmall $Fe_3O_4$ NPs.

The activated USIO NPs (1.48, 2.96, 5.92 or 8.88 mg) were respectively added into the Au DSNP solution (28 mg, 10 mL in water) and the reaction was continued for 3 days under magnetic stirring to acquire the $Fe_3O_4$/Au DSNPs complexes with different Fe/Au molar ratios. After that, the product was dialyzed against water (6 times, 2 L) using a dialysis membrane with an MWCO of 3000 for 3 days to purify the $Fe_3O_4$/Au DSNPs complexes. The final $Fe_3O_4$/Au DSNPs complexes were dispersed into 10 mL of water before use.

Formation of $Fe_3O_4$/Au DSNFs

The above $Fe_3O_4$/Au DSNPs complexes (100 μL) with different Fe/Au molar ratios were separately added into an aqueous solution of $HAuCl_4$ (0.25 mM, 10 mL) under stirring, then $AgNO_3$ (2 mM, 100 μL in water) and AA (0.1 M, 50 μL in water) were immediately added

into the above solution. The solution was stirred for 2 h to form Au DSNFs embedded with USIO NPs. After that, the remaining dendrimer terminal amine groups on the surface of the Au DSNFs were completely acetylated according to the literature.[3] Briefly, 3.8 μL of triethylamine was added to the aqueous solution of $Fe_3O_4$/Au DSNFs, and the solution was adequately mixed for 30 min. Then, 2.1 μL of acetic anhydride was added into the above solution under vigorous magnetic stirring for 24 h. Finally, the above mixture was centrifuged at 8500 rpm for 20 min and redispersed into water for at least three times and the purified particles were lyophilized to obtain the final $Fe_3O_4$/Au DSNFs. For comparison, Au DSNFs without USIO NPs were also prepared under the same conditions.

To optimize the final product of the $Fe_3O_4$/Au DSNFs, we used $Fe_3O_4$/Au DSNPs complexes with different Fe/Au molar ratios (1 : 1 to 6 : 1) as seeds and assessed the colloidal stability, size, surface potential and NIR absorption properties of the as-synthesized $Fe_3O_4$/Au DSNFs to optimize the Fe/Au molar ratio for the product of $Fe_3O_4$/Au DSNFs.

Characterization Techniques

Transmission electron microscopy (TEM) was carried out by JEOL 2010F electron microscope (Tokyo, Japan) at an operating voltage of 200 kV. A typical sample with a volume 6 μL in water was deposited onto carbon-coated copper grid and air dried before observation. Dynamic light scattering (DLS) and zeta potential measurements were performed using a Malvern Zetasizer (Nano ZS model ZEN3600, Worcestershire, UK) equipped with a standard 633 nm laser. Samples were dispersed in water with a concentration of 1 mg/mL before measurements. Thermal gravimetric analysis (TGA) was executed using a TG 209 F1 thermal gravimetric analyzer (NETZSCH Instruments Co., Ltd., Selb/Bavaria, Germany) at a heating rate of 20 $^{o}$C/min and in a temperature range of 30-700 $^{o}$C under $N_2$ atmosphere. UV-vis spectra were collected using a Lambda 25 UV-vis spectrophotometer (Perkin Elmer, Boston, MA). Each sample was dispersed in water and placed into a cuvette (1 mL) before

measurements. X-ray diffraction (XRD) analysis was carried out using a D/max 2550 VB+/PC X-ray diffractometer (Rigaku Cop., Tokyo, Japan) with Cu Kα radiation ($\lambda$ = 0.154056 nm) at 40 kV and 200 mA and a 2θ scan range of 5-90°. The Fe and Au composition of the formed USIO NPs, Au DSNPs, $Fe_3O_4$/Au DSNPs complexes or $Fe_3O_4$/Au DSNFs was determined by Leeman Prodigy inductively coupled plasma-optical emission spectroscopy (ICP-OES, Hudson, NH).

Photothermal Property of the $Fe_3O_4$/Au DSNFs

To assess the concentration-dependent photothermal conversion property of the $Fe_3O_4$/Au DSNFs, the particles with different Au concentrations (0, 1, 2, 5, 10 or 20 mM, respectively) were put into a quartz cuvette, and then irradiated by an 808 nm laser for 300 s (facula area: 0.25 $cm^2$, output power density: 1.2 $W/cm^2$) using a laser device (Shanghai Xilong Optoelectronics Technology Co. Ltd., Shanghai, China). A DT-8891E thermocouple thermometer (Shenzhen Everbest Machinery Industry Co., Ltd., Shenzhen, China) was used to record the in situ temperature change of different samples every 5 s. Au DSNFs ([Au] = 1 mM) were also tested for comparison. The photothermal conversion efficiency ($\eta$) of the $Fe_3O_4$/Au DSNFs and the Au DSNFs was then calculated according to the literature.[4] The photothermal stability of the $Fe_3O_4$/Au DSNFs was testified via comparing the temperature change with or without 808 nm NIR laser irradiation (1.2 $W/cm^2$) for five cycles.[5]

MR/CT/PA/Thermal Imaging Performance of the $Fe_3O_4$/Au DSNFs

$T_1$ magnetic resonance (MR) relaxometry was performed by a 0.5 T NMI20 Analyzing and Imaging system (Shanghai NIUMAG Corporation, Shanghai, China). The parameters were set as follows: TR = 400 ms, TE = 20 ms, resolution = 156 mm × 156 mm, and section thickness = 0.5 mm. The $r_1$ relaxivity was obtained through linear fitting of the inverse $T_1$ relaxation time ($1/T_1$) as a function of Fe concentration. Both USIO NPs and $Fe_3O_4$/Au DSNFs with the same Fe concentrations (0.1-1.6 mM) were determined. Concentration-dependent brightening effects were observed by $T_1$-weighted MR images.

GE LightSpeed VCT imaging system (GE Medical Systems, Milwaukee, WI) was applied for CT scanning at 100 kV, 80 mA, and a slice thickness of 0.625 mm. Contrast enhancement was determined in Hounsfield units (HU) for each sample with different Au concentrations. The CT imaging performance of $Fe_3O_4$/Au DSNFs was compared to clinical contrast agent (Omnipaque) at different molar concentrations of Au or iodine (2.5-40 mM).

For PA imaging, each individual hole on a home-made agar plate was filled with 200 μL of $Fe_3O_4$/Au DSNFs at different Au concentrations (0-40 mM). The samples were monitored using Vevo® LAZR photoacoustic imaging system (VisualSonics Inc., Toronto, Canada) with an laser of 808 nm (excitation wavelength).

For thermal imaging, the PBS (control) or PBS solution of $Fe_3O_4$/Au DSNFs (1 mL, [Au] = 2 mM) was placed into a cuvette (1.5 mL). Then, the sample was irradiated by an 808 nm NIR laser (1.2 W/cm$^2$) for 300 s and the thermal images were recorded at different time points (0, 1, 2, 3, 4 and 5 min, respectively) according to the literature protocols.[5]

In Vitro Cytotoxicity Assay

To evaluate the intrinsic cytotoxicity of the prepared $Fe_3O_4$/Au DSNFs, CCK-8 assay of 4T1 cells treated with the $Fe_3O_4$/Au DSNFs at different concentrations ([Au] = 0-2.0 mM) was performed according to protocols described in the literature.[6] 4T1 cells were first cultured in a 25 cm$^2$ cell culture flask with DMEM supplemented with 10% FBS and 1% penicillin/streptomycin under 37 $^{o}$C and 5% $CO_2$. After that, 4T1 cells were seeded into a 96-well plate with 100 μL of fresh DMEM at a density of 8 × 10$^3$ cells/well and cultured at 37 °C and 5% $CO_2$ overnight. The medium was replaced with fresh medium containing $Fe_3O_4$/Au DSNFs with different Au concentrations for 24 h. Then CCK-8 (10 μL) was added into each well and the cells were incubated for another 3 h. The absorbance of each well was recorded using a Thermo Scientific Multiskan MK3 ELISA reader (Thermo Scientific, Waltham, MA) at 450 nm.

Cellular Uptake of $Fe_3O_4$/Au DSNFs

The cellular uptake of the $Fe_3O_4$/Au DSNFs was then assessed via ICP-OES. 4T1 cells were seeded in 12-well plates at a density of $2 \times 10^5$ cells per well in 1 mL of DMEM and incubated at 37 °C and 5% $CO_2$. After overnight incubation, the medium was replaced with 1 mL of fresh medium containing PBS (control) and $Fe_3O_4$/Au DSNFs at different Au concentrations (0.2-2.0 mM), and the cells were incubated for another 6 h. Afterwards, the cells were washed with PBS for 3 times, trypsinized, centrifuged, and resuspended in 1 mL of PBS for counting the cell numbers. Subsequently, the cells were digested by aqua regia solution (1.0 mL) overnight, and diluted with 1.0 mL of water, followed by ICP-OES assay to determine the Au content in the cell samples.

To further confirm the cellular uptake of the $Fe_3O_4$/Au DSNFs, Prussian blue staining was employed.[1] Similar to the ICP-OES analysis, 4T1 cells were treated with the $Fe_3O_4$/Au DSNFs at different Fe concentrations (0.44-1.75 mM) for 6 h, then the cells were washed 3 times with PBS, and stained by Prussian blue solution. Afterwards, the stained cells were observed by Leica DM IL LED inverted phase contrast microscope.

Photothermal Ablation of Cancer Cells in Vitro

CCK-8 and Live-Dead Cell Staining Kit were used to characterize the photothermal ablation of 4T1 cells treated with the $Fe_3O_4$/Au DSNFs. 4T1 cells at a density of $8 \times 10^3$ cells per well in 100 μL medium were seeded into a 96-well plate and cultured at 37 °C and 5% $CO_2$ overnight. After that, the medium was replaced with 100 μL of fresh medium containing PBS (control) and $Fe_3O_4$/Au DSNFs (0.2, 0.4, 0.6, 0.8, 1.0 or 2.0 mM, respectively), and the cells were incubated for 4 h. After being rinsed with PBS for 3 times, the cells were irradiated by an 808 nm NIR laser (1.2 W/cm$^2$, 5 min). After incubation of the cells for an additional 3 h, the cell viability was evaluated by CCK-8 assay.

Calcein AM (green) and propidiumiodide (PI, red) co-staining was used to further qualitatively illustrate the photothermal killing effect of $Fe_3O_4$/Au DSNFs through distinguishing live and dead cells. 4T1 cells with a density of $1 \times 10^5$ cells per well in 1 mL of

medium were seeded into 24-well plate. After overnight incubation, the medium of cells was replaced with fresh medium containing PBS or $Fe_3O_4$/Au DSNFs at the Au concentration of 2.0 mM, respectively. After 6 h incubation, the medium was discarded and the cells were rinsed with PBS for 3 times and incubated with fresh medium, then irradiated with an 808 nm laser (1.2 W/cm$^2$, 5 min). After that, the cells were stained with calcein-AM and PI according to the standard procedures.[7] The cells were observed using an Axio Vert. A1 inverted fluorescence microscope (Carl Zeiss, Jena, Germany) with a magnification of 100 × for each sample.

In Vivo MR/CT/PA Imaging of Tumors

We performed animal experiments following the protocols approved by the institutional committee for animal care and the policy of the National Ministry of Health. Male nude mice (15-20 g, Shanghai Slac Laboratory Animal Center, Shanghai, China) were subcutaneously injected with 5 × 10$^6$ 4T1 cells/mouse in the left back. When the tumor nodules reached a volume of about 0.1 cm$^3$, the mice were anesthetized by intraperitoneal injection of pentobarbital sodium (40 mg/kg for each mouse), then the $Fe_3O_4$/Au DSNFs dispersed in 200 μL of normal saline (NS, [Fe]= 5.5 mM, and [Au] = 40 mM) were injected into each tumor-bearing mouse via the tail vein. The mice were scanned by a 3.0 T clinical MR imaging system (SOMATON Definition Flash, Siemens, Erlangen, Germany). The parameters of MR imaging were set as follows: TR = 300, 600, 900, 1200 ms, TE = 10.7 ms, matrix = 256 × 256, section thickness = 2 mm, and FOV = 12 cm. $T_1$-weighted MR images were obtained before and after intravenous injection of the USIO NPs and $Fe_3O_4$/Au DSNFs at the time points of 0, 15, 30, 45, 60, 75 and 90 min, respectively.

For in vivo CT imaging, the $Fe_3O_4$/Au DSNFs ([Fe]= 5.5 mM, [Au] = 40 mM, in 200 μL NS) were injected into each mouse via the tail vein. CT images of tumors were collected at different time points (0, 15, 30, 45, 60, 75 and 90 min, respectively) post intravenous injection

by a GE LightSpeed VCT imaging system with the parameters similar to those mentioned above. The tumor CT values were then quantified.

In vivo PA imaging of tumors was investigated by a Vevo LAZR PA Imaging System under an 808 nm laser irradiation. $Fe_3O_4$/Au DSNFs ([Fe]= 5.5 mM, [Au] = 40 mM, in 200 μL NS) were injected to each mouse via tail vein. The PA images and signal intensity of the tumor site were obtained using the PA imaging system before and at 15, 30, 45, 60, 75, or 90 min post intravenous injection of the $Fe_3O_4$/Au DSNFs.

In Vivo Thermal Imaging of Tumors

In vivo thermal imaging was undertaken by an infrared camera. The $Fe_3O_4$/Au DSNFs ([Au] = 40 mM, in 200 μL NS) were intratumorally injected to each mouse. NS was used as control. Then, the tumor region was irradiated by an 808 nm NIR laser for 5 min and the thermal images were recorded according to the literature protocols.[5]

In Vivo PTT/RT Combination Therapy

Mice bearing 4T1 xenograft tumors were divided into 6 groups (5 mice for each group): (1) NS; (2) RT alone; (3) NFs alone; (4) NFs + Laser; (5) NFs + RT; and (6) NFs + Laser + RT. Mice were injected intratumorally with the $Fe_3O_4$/Au DSNFs (in 100 μL NS, [Au] = 20 mM for each mouse for the related groups). After 24 h, laser irradiation was performed using an 808 nm laser with an output power density of 1.2 $W/cm^2$ for 5 min (laser groups), and radiation treatment was carried out under 4 Gy of X-ray radiation (for RT groups). Body weight and tumor volume of each mouse were measured for 15 days (n = 5), while survival rates were also measured as the percentages of surviving mice compared to total mice for each group (n = 5).

H&E and TUNEL Staining

To further evaluate the potential toxicity of the $Fe_3O_4$/Au DSNFs in vivo, the mice were intravenously injected with the particles (20 mM, in 100 μL NS for each mouse) via tail vein.

At 14 days, major organs including liver, heart, lung, kidney, spleen, and tumors were extracted from euthanized mice and weighed. After being fixed in 4% paraformaldehyde, paraffin-embedded tissues were sectioned for hematoxylin and eosin (H&E) staining and then observed under a Leica DM IL LED inverted phase contrast microscope.

To study the mechanism of in vivo tumor photothermal ablation and the effect of RT and PTT/RT, the 4T1 tumor-bearing mice in each group were euthanized at 4 h posttreatment. The tumors were then extracted and processed according the above protocols for H&E and TdT-mediated dUTP Nick-End Labeling (TUNEL) staining using standard protocols.[5] The morphology of tumor sections of each group was observed using a Leica DM IL LED inverted phase contrast microscope and the tumor cell apoptosis rate was quantified. The percentage of TUNEL-positive cells in each sample was determined from five random selected fields.

In Vivo Biodistribution

The biodistribution of $Fe_3O_4$/Au DSNFs was investigated in the tumor-bearing mice via ICP-OES. At different time points (1 h, 24 h and 96 h, respectively) post intravenous injection of $Fe_3O_4$/Au DSNFs ([Au] = 0.04 M, in 200 μL PBS for each mouse), the mice were sacrificed and the major organs (heart, liver, spleen, lung and kidney) and tumor were extracted, weighed, digested by aqua regia, and diluted with water. The content of Au element in different organs was determined by ICP-OES, and the results were represented as the amount of Au (μg) per gram of wet tissue. The mice injected with PBS (200 μL for each mouse) were used as control.

Statistical analysis

One-way analysis of variance (ANOVA) statistical method was performed to evaluate the experimental data. A value of 0.05 was selected as the significance level and the data were indicated with (*) for $p < 0.05$, (**) for $p < 0.01$, and (***) for $p < 0.001$, respectively.

**References**

[1] D. Ma, J. W. Chen, Y. Luo, H. Wang and X. Y. Shi, J. Mater. Chem. B **2017**, 5, 7267-7273.
[2] H. Liu, Y. H. Xu, S. H. Wen, J. Y. Zhu, L. F. Zheng, M. W. Shen, J. L. Zhao, G. X. Zhang and X. Y. Shi, Polym. Chem. **2013**, 4, 1788-1795.
[3] J. Y. Zhu, L. F. Zheng, S. H. Wen, Y. Q. Tang, M. W. Shen, G. X. Zhang and X. Y. Shi, Biomaterials **2014**, 35, 7635-7646.
[4] a) Y. Hu, R. Z. Wang, S. G. Wang, L. Ding, J. C. Li, Y. Luo, X. L. Wang, M. W. Shen and X. Y. Shi, Sci. Rep. **2016**, 6, 27325015 ; b) Y. Zhou, Y. Hu, W. Sun, S. Lu, C. Cai, C. Peng, J. Yu, R. Popovtzer, M. Shen and X. Shi, Biomacromolecules **2018**, 19, 2034-2042.
[5] X. Li, L. X. Xing, K. L. Zheng, P. Wei, L. F. Du, M. W. Shen and X. Y. Shi, ACS Appl. Mater. Interfaces **2017**, 9, 5817-5827.
[6] J. Chen, Z. H. Sheng, P. H. Li, M. X. Wu, N. S. Zhang, X. F. Yu, Y. W. Wang, D. H. Hu, H. R. Zheng and G. P. Wang, Nanoscale **2017**, 9, 11888-11901.
[7] Y. W. Zhou, Y. Hu, W. J. Sun, B. Q. Zhou, J. Z. Zhu, C. Peng, M. W. Shen and X. Y. Shi, Nanoscale **2017**, 9, 12746-12754.

**Supplementary Figures**

**Table S1.** Zeta potential and hydrodynamic size of the USIO NPs, Au DSNPs, $Fe_3O_4$/Au DSNPs and $Fe_3O_4$/Au DSNFs (after acetylation) with different molar ratios of Fe/Au, respectively.

| Sample | Zeta Potential (mV) | Hydrodynamic Size (nm) | PDI |
|---|---|---|---|
| USIO NPs | -26.8 ± 2.8 | 27.5 ± 3.2 | 0.162 ± 0.058 |
| Au DSNPs | 24.6 ± 0.2 | 37.7 ± 8.9 | 0.250 ± 0.085 |
| $Fe_3O_4$/Au DSNPs (1 : 1) | 12.5 ± 1.7 | 26.9 ± 4.9 | 0.501 ± 0.076 |
| $Fe_3O_4$/Au DSNPs (2 : 1) | 11.4 ± 0.9 | 27.5 ± 2.1 | 0.107 ± 0.007 |
| $Fe_3O_4$/Au DSNPs (4 : 1) | 10.8 ± 2.4 | 28.2 ± 2.3 | 0.416 ± 0.057 |
| Au DSNFs | 16.9 ± 0.8 | 114.6 ± 5.5 | 0.172 ± 0.028 |
| $Fe_3O_4$/Au DSNFs (1 : 1) | 16.1 ± 1.2 | 193.4 ± 13.6 | 0.302 ± 0.079 |
| $Fe_3O_4$/Au DSNFs (2 : 1) | 15.4 ± 0.6 | 285.2 ± 5.6 | 0.225 ± 0.034 |
| $Fe_3O_4$/Au DSNFs (4 : 1) | 14.7 ± 1.0 | 302.2 ± 8.0 | 0.472 ± 0.056 |

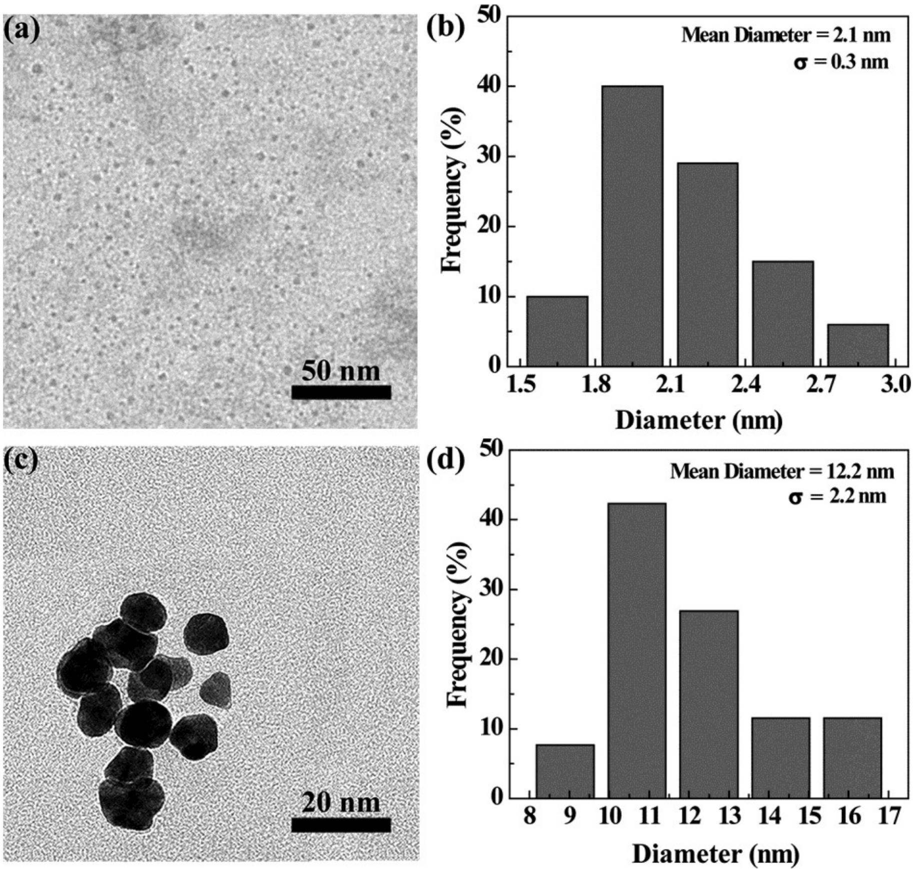


**Figure S1**. TEM images (a, c) and size distribution histogram (b, d) of USIO NPs (a, b) and Au DSNPs (c, d), respectively.

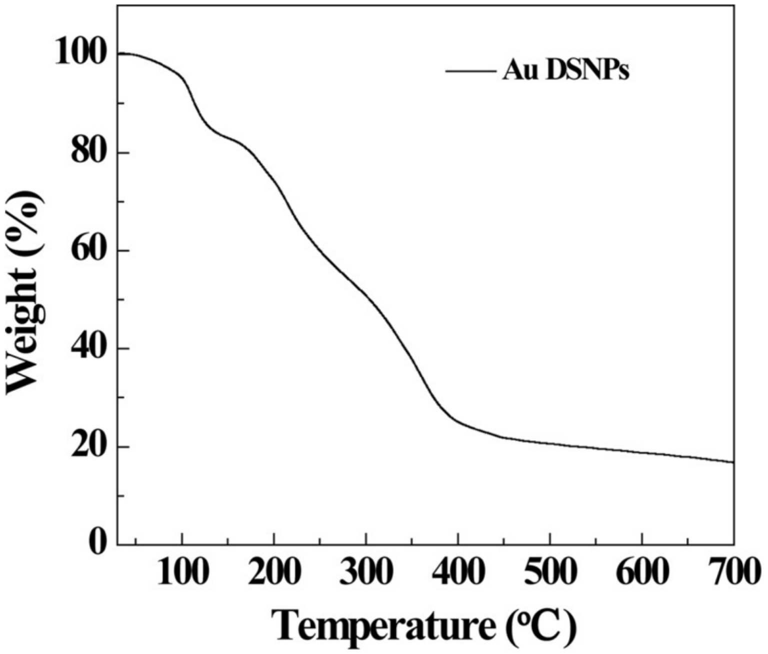


**Figure S2**. TGA curve of Au DSNPs.

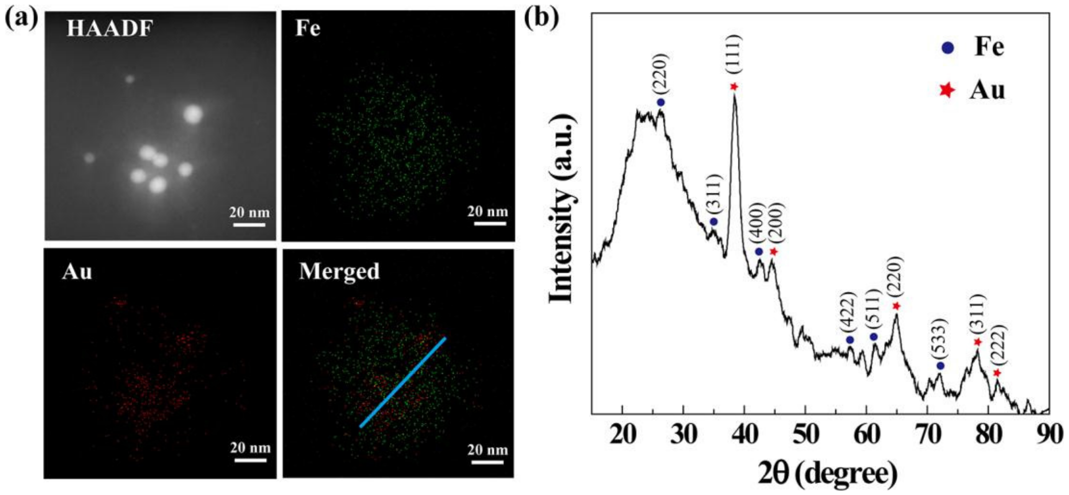


**Figure S3**. Element mapping (a) and XRD pattern (b) of $Fe_3O_4$/Au DSNPs.

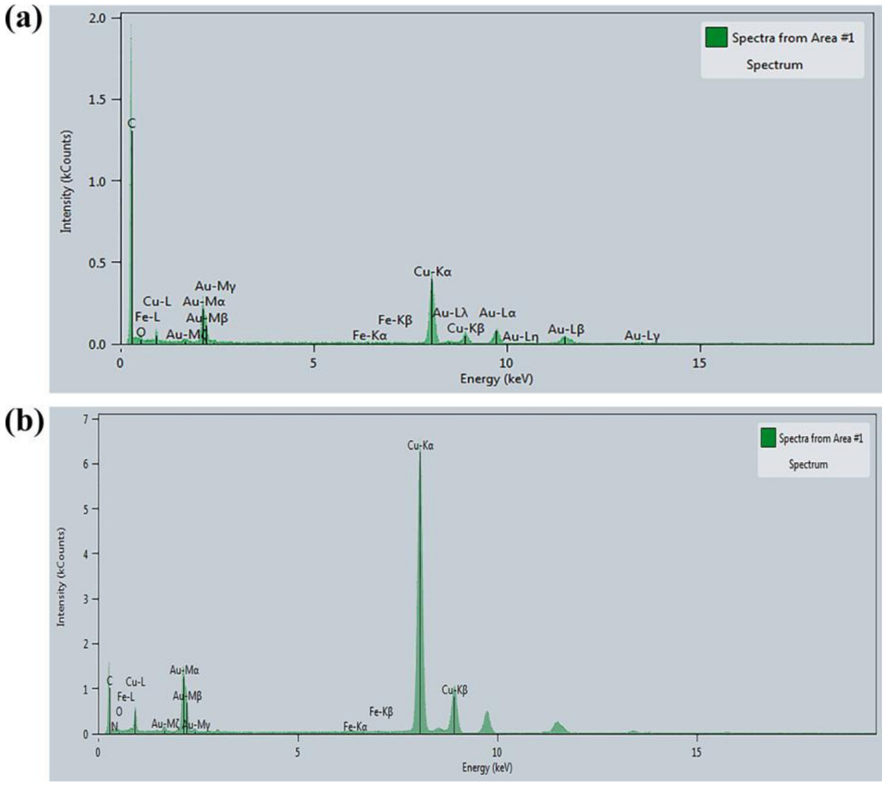


**Figure S4**. Energy-dispersive spectroscopy (EDS) line scanning of $Fe_3O_4$/Au DSNPs (a) and $Fe_3O_4$/Au DSNFs (b), respectively.

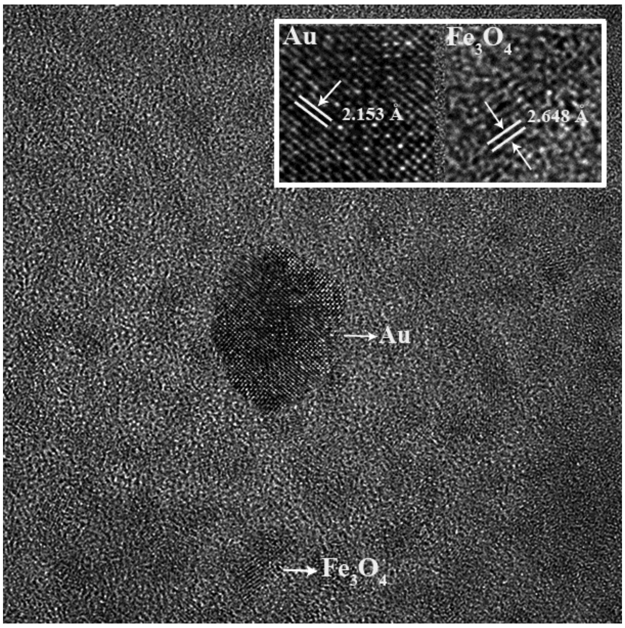


**Figure S5**. The interplanar crystal spacing of Au and USIO NPs in the $Fe_3O_4$/Au DSNPs.

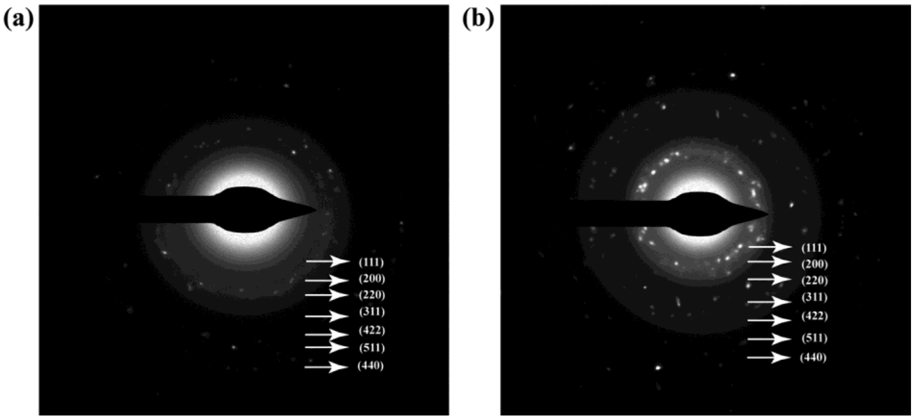


**Figure S6.** The selected area electron diffraction pattern of $Fe_3O_4$/Au DSNPs (a) and $Fe_3O_4$/Au DSNFs (b).

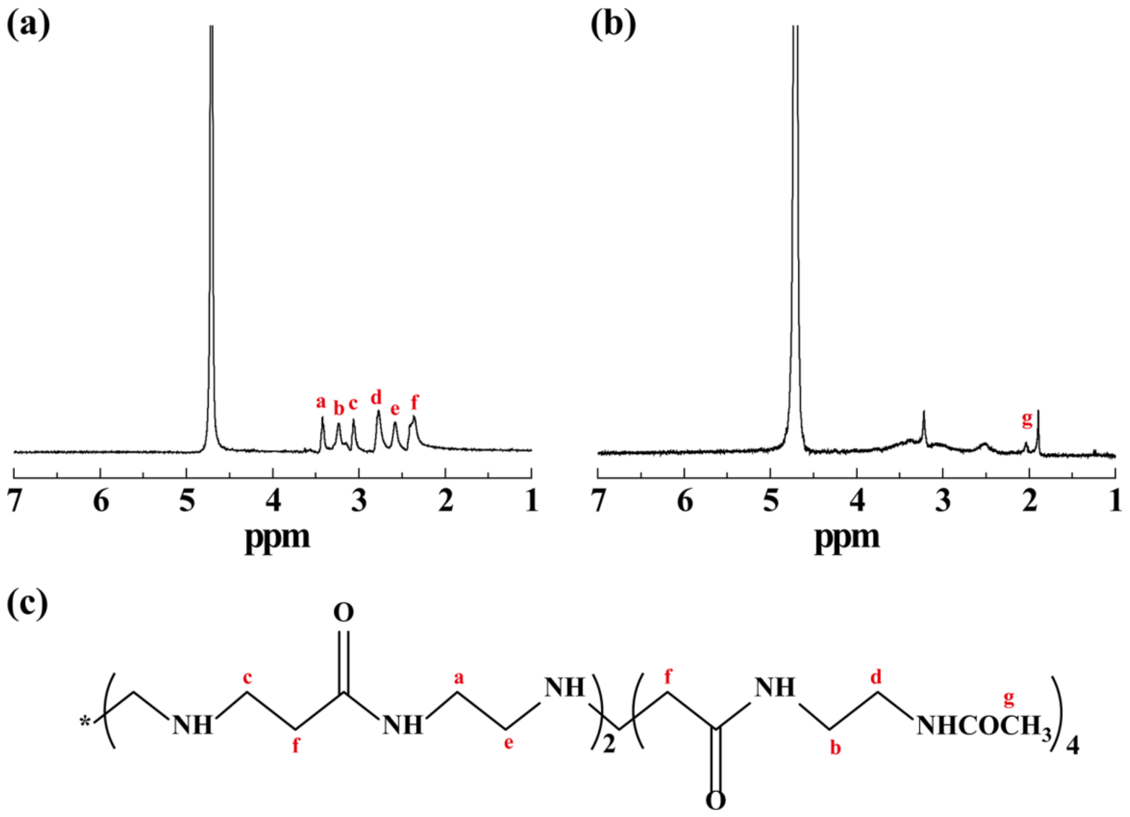


**Figure S7**. $^{1}$H NMR spectra of $Fe_3O_4$/Au DSNFs dispersed in $D_2O$ before (a) and after (b) acetylation. (c) shows the molecular structure of acetylated G5 dendrimers.

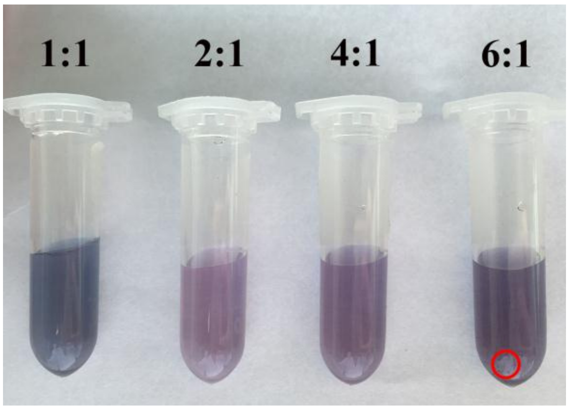


**Figure S8**. Digital photos of $Fe_3O_4$/Au DSNFs with different molar ratios of Fe/Au (from 1:1 to 6:1).

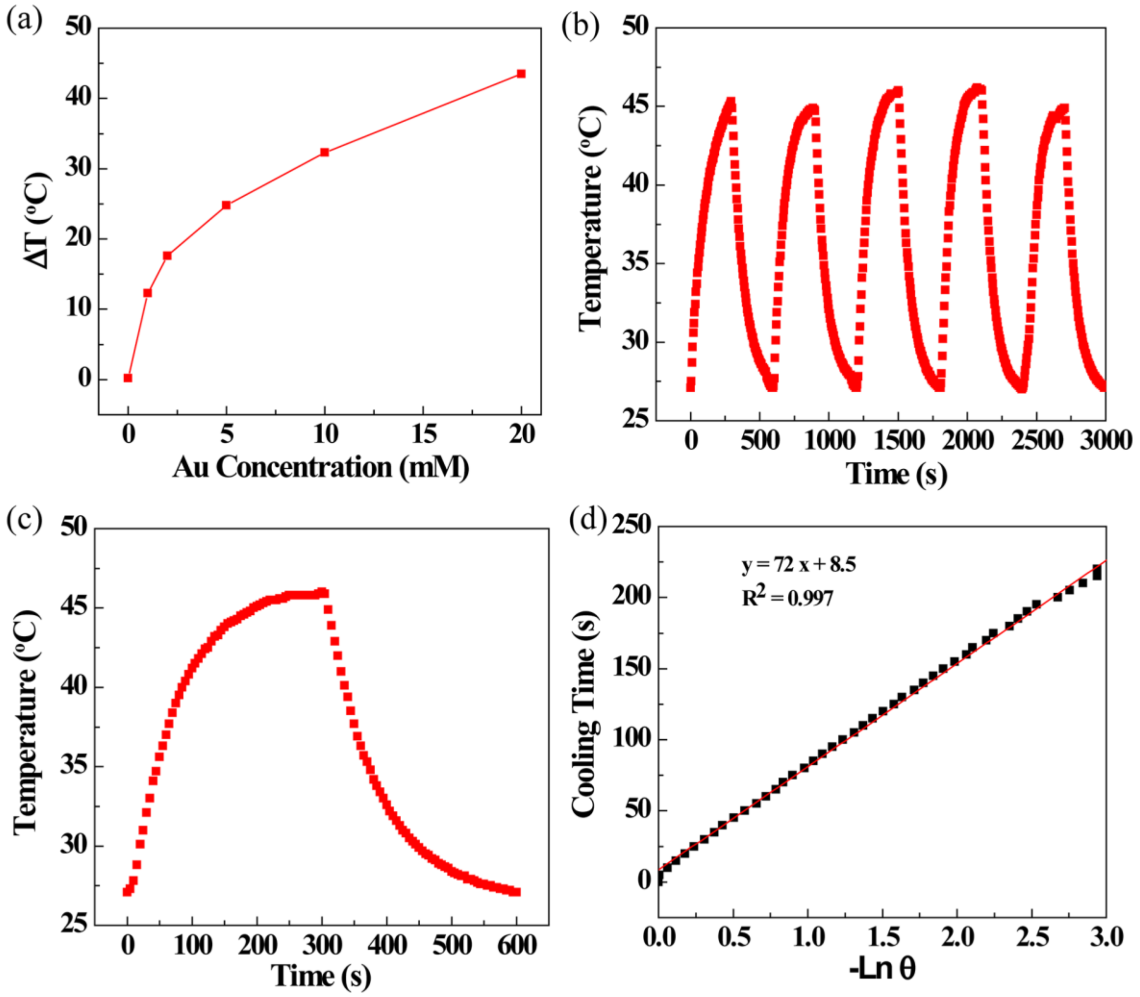


**Figure S9.** (a) The temperature change (ΔT) of the aqueous solution of the $Fe_3O_4$/Au DSNFs at different Au concentrations over a period of 300 s. (b) Temperature plot of the aqueous solution containing the $Fe_3O_4$/Au DSNFs ([Au] = 2 mM) as a function of time (laser on for 300 s and laser off for each cycle) for five cycles. (c) Plot of the temperature vs time for the $Fe_3O_4$/Au DSNFs (2 mM, $OD_{808}$ = 1.403) during laser irradiation (808 nm, 1.2 W/cm$^2$) and cooling (laser off) stages. (d) Plot of the cooling time vs -lnθ. On the basis of the linear fitting analysis, the time constant for heat transfer $\tau_s$ (the slope of the plot) was determined to be 72 s.

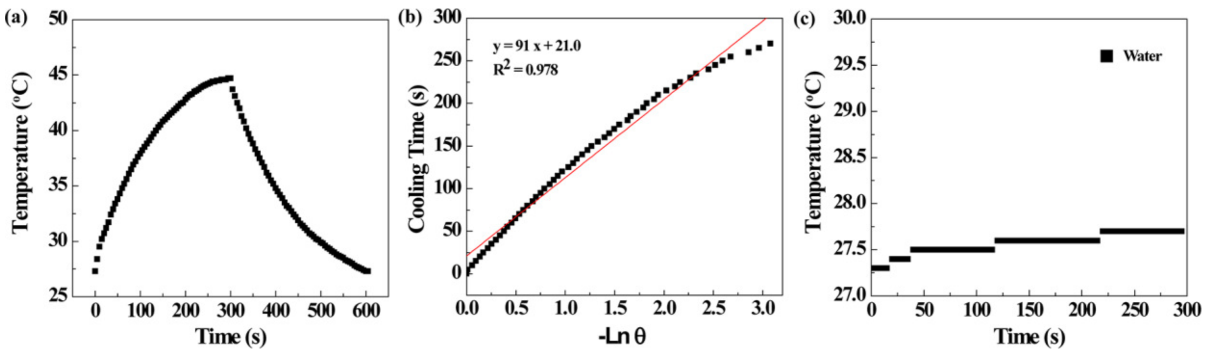


**Figure S10.** (a) Plot of the temperature vs time for the Au DSNFs (2 mM, $OD_{808}$ = 2.045) during laser irradiation (808 nm, 1.2 W/cm$^2$) and cooling (laser off) stages. (b) Plot of the cooling time vs -lnθ. On the basis of the linear fitting analysis, the time constant for heat transfer $\tau_s$ (the slope of the plot) was determined to be 91 s. (c) Plot of the temperature vs time for pure water during laser irradiation (808 nm, 1.2 W/cm$^2$).

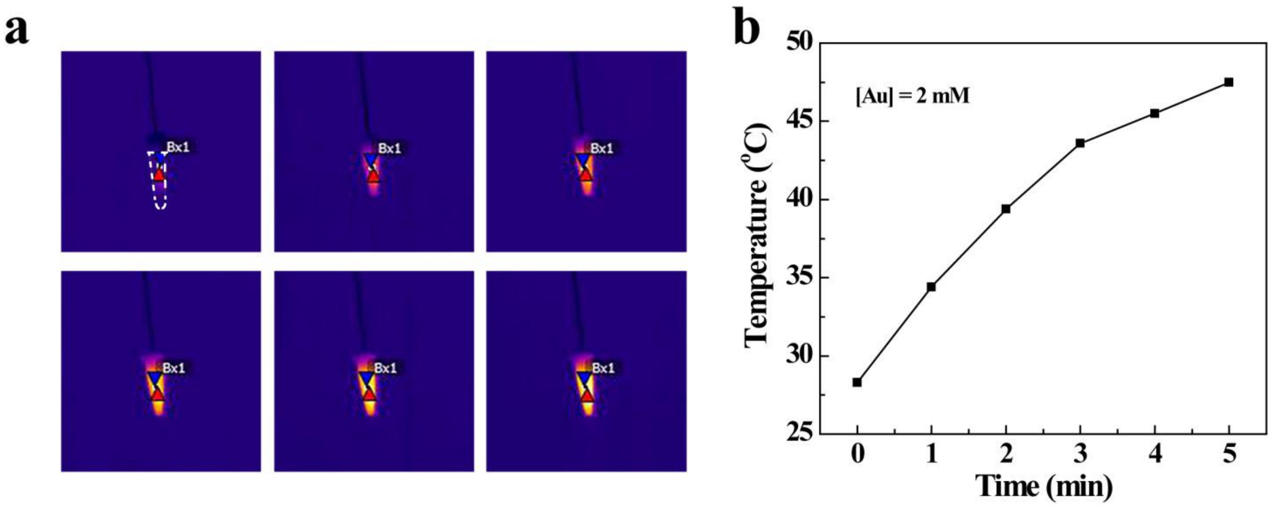


**Figure S11.** Thermal images (a) and correspond temperature profiles (b) of $Fe_3O_4$/Au DSNFs aqueous solution ([Au] = 2 mM, 1 mL) irradiated with an 808 nm laser (1.2 W/cm$^2$) at the time points of 0, 1, 2, 3, 4 and 5 min, respectively.

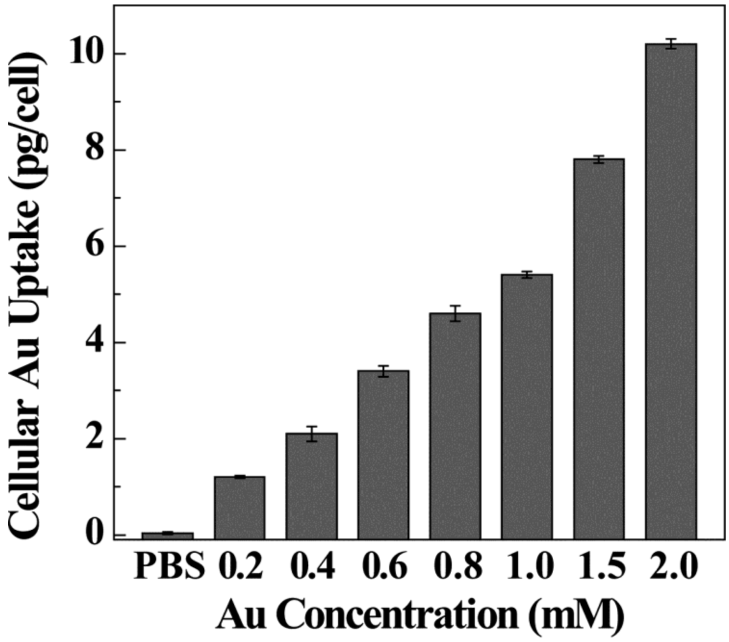


**Figure S12.** The Au uptake by 4T1 cells after treated with the $Fe_3O_4$/Au DSNFs at various Au concentrations for 6 h.

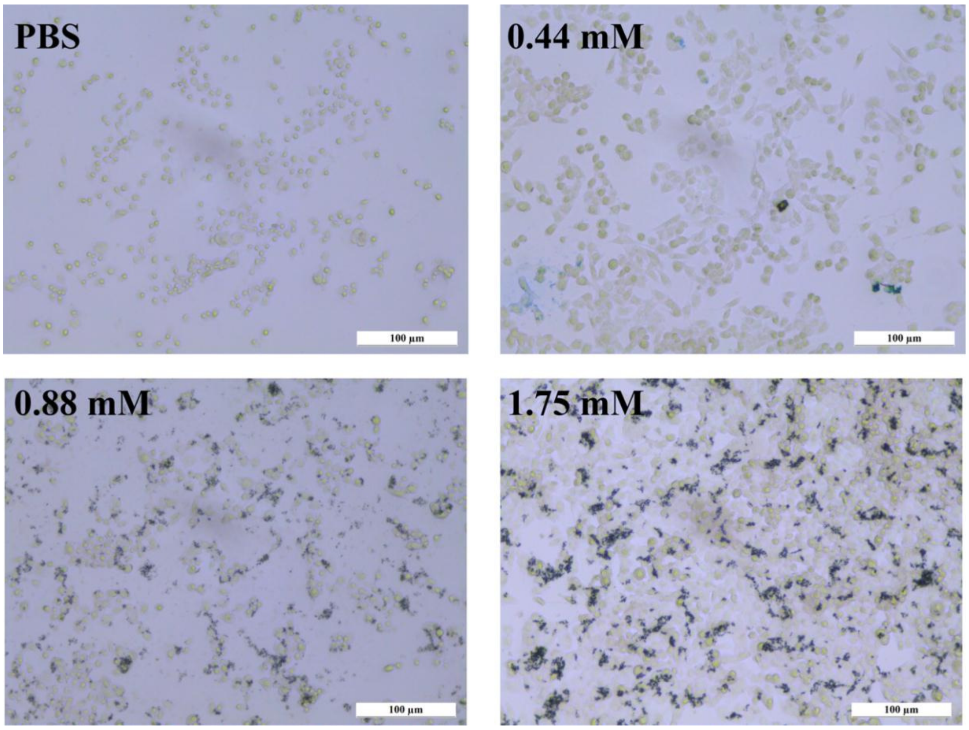


**Figure S13.** Prussian blue staining of 4T1 cells treated with the $Fe_3O_4$/Au DSNFs. 4T1 cells treated with PBS were used as control. Cells were treated with the $Fe_3O_4$/Au DSNFs at an Fe concentration of 0.44, 0.88, or 1.75 mM. In all cases, the incubation time was 6 h. Blue staining indicates the presence of iron element. The scale bar in each panel represents 100 μm.

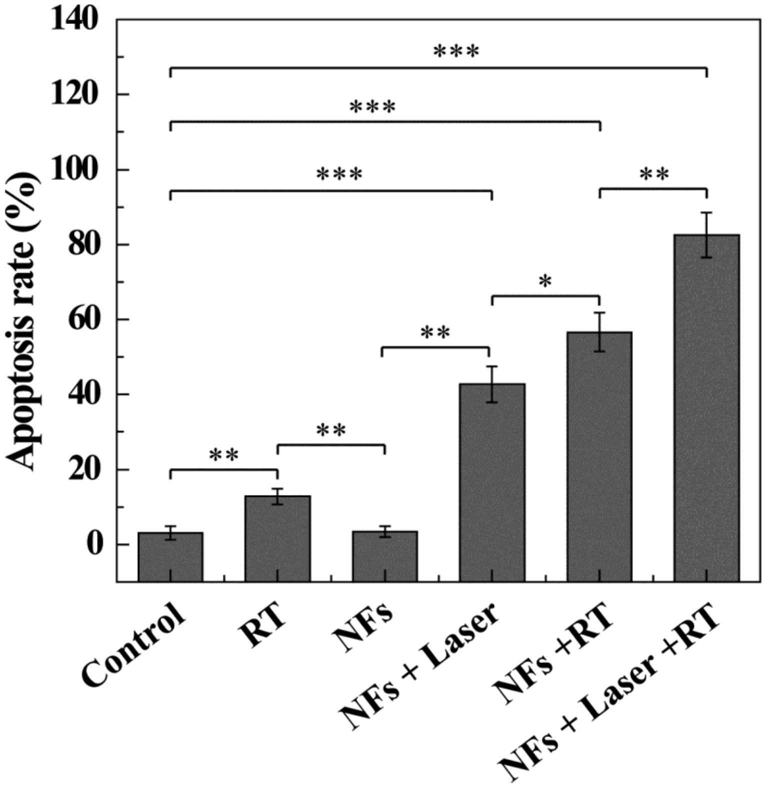


**Figure S14**. Apoptosis rate of tumor cells after different treatments by quantification of the TUNEL-positive tumor cells in random tumor sections.

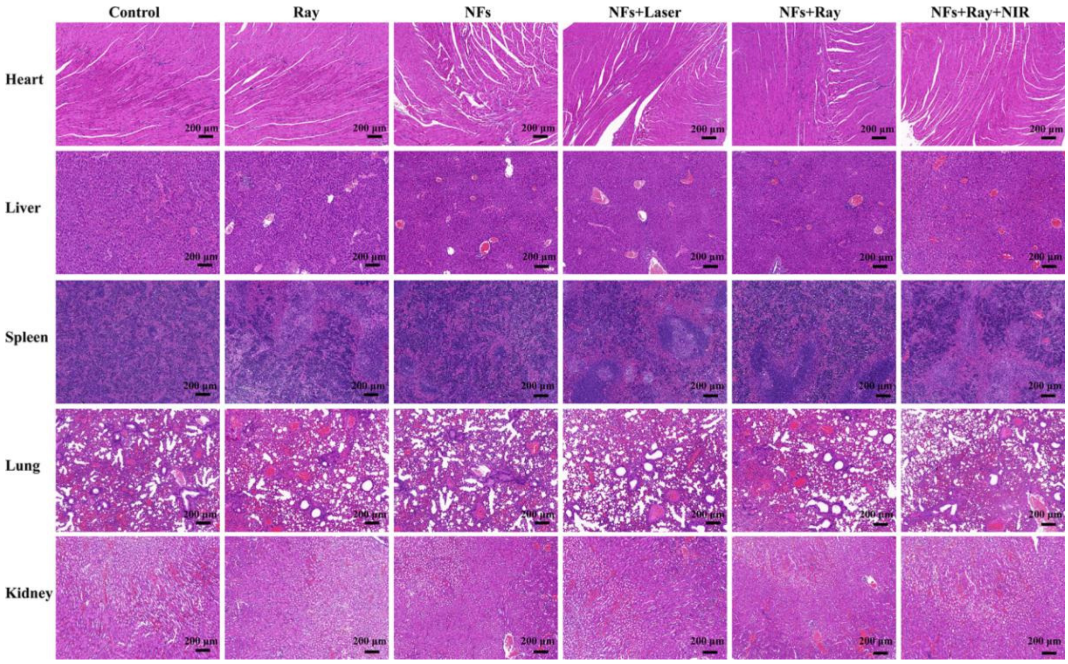


**Figure S15.** H&E-stained tissue sections of mice at 20 days post intravenous injection of the $Fe_3O_4$/Au DSNFs ([Au] = 20 mM, in 0.2 mL NS for each mouse). The mice treated with NS were used as the control. The scale bar shown in each panel represents 200 μm.

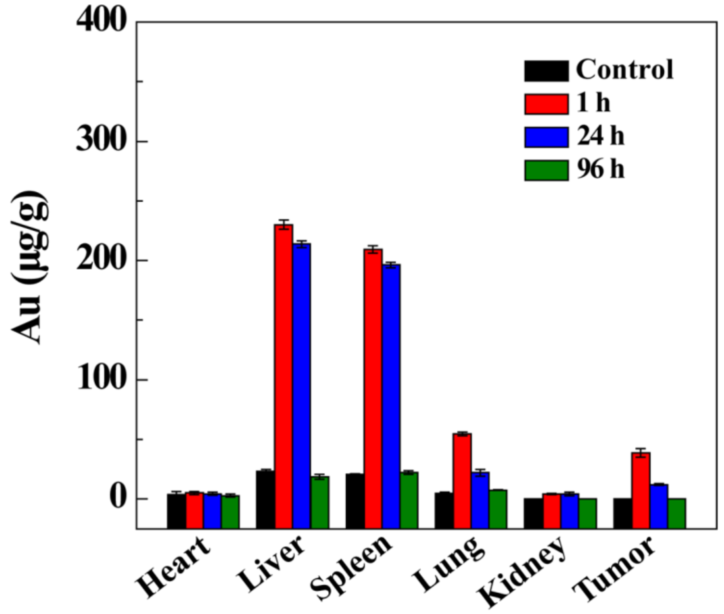


**Figure S16.** In vivo biodistribution of Au element in different organs and tumor at 1 h, 24 h and 96 h post intravenous injection of the $Fe_3O_4$/Au DSNFs ([Au] = 0.04 M, in 200 μL PBS for each mouse). Mice injected with PBS (200 μL for each mouse) were used as control.
